# Aging in Precarious Place: How Older Adults Navigate Disaster Recovery and Negotiate Climate Risk

**DOI:** 10.1093/geroni/igaf122.791

**Published:** 2025-12-31

**Authors:** Alexis Merdjanoff

**Affiliations:** New York University, New York, New York, United States

## Abstract

Community-dwelling older adults frequently age in states that are susceptible to various disasters, such as hurricanes, heat waves, wildfires, blizzards, and flooding. Despite their vulnerability, there has been little planning focused on providing community-dwelling older adults with the resources they need to prepare for and recover from climate-related disasters. This presentation will use Hurricane Ian as a case study to understand the social resources that older adults need to recover from disaster and age in precarious place. Hurricane Ian was responsible for the direct and indirect deaths of 150 Floridians and caused over $112 billion in damage, making it the costliest hurricane in Florida’s history and the third-costliest in U.S. history. Analyses includes 73 in-depth interviews with older adults who were living in Southwest Florida at the time of the storm, and collected between September 2023 and November 2023. This presentation will examine three themes that have been identified using a grounded theory approach: 1) homeowners’ insurance as a barrier to aging in place; 2) adult children as a key facilitator of older adult recovery; and, 3) a commitment to aging in precarious place. This study’s initial themes raise questions regarding the social infrastructure needs of older adult communities that are at-risk for climate-related disasters. This presentation will conclude by addressing the types of local programming, community non-profits and public spaces that older adults need in order to age well, despite the climate crisis.

